# Theaflavin-3,3′-Digallate Protects Cartilage from Degradation by Modulating Inflammation and Antioxidant Pathways

**DOI:** 10.1155/2022/3047425

**Published:** 2022-07-08

**Authors:** Yun Teng, Zheyu Jin, Weizhi Ren, Minghao Lu, Mingzhuang Hou, Quan Zhou, Wenhao Wang, Huilin Yang, Jun Zou

**Affiliations:** ^1^Department of Orthopaedic Surgery, The First Affiliated Hospital of Soochow University, Suzhou, Jiangsu, China; ^2^Department of Orthopaedic Surgery, The Second Affiliated Hospital of Soochow University, Suzhou, Jiangsu, China; ^3^Department of Orthopaedic Surgery, The Second People's Hospital of Changshu, Suzhou, Jiangsu, China

## Abstract

**Background:**

Osteoarthritis (OA) is a common degenerative joint disease that may be closely linked to inflammation and oxidative stress destroying the balance of cartilage matrix. Theaflavin-3,3′-digallate (TFDG), a natural substance derived from black tea, has been reported to restrict the activity of inflammatory cytokines and effectively eliminate reactive oxygen species (ROS) in various diseases. However, it is not clear whether TFDG can improve OA.

**Methods:**

Chondrocytes were treated with or without IL-1*β* and 20 *μ*M and 40 *μ*M TFDG. The effect of TFDG on the proliferation of chondrocytes was detected by CCK8. RT-qPCR was used to detect the gene expression of inflammatory factors, extracellular matrix synthesis, and degradation genes. Western blot and immunofluorescence assays were used to detect the protein expression. The fluorescence intensity of reactive oxygen species labeled by DCFH-DA was detected by flow cytometry. We established an OA rat model by performing destabilized medial meniscus (DMM) surgery to observe whether TFDG can protect chondrocytes under arthritis in vivo.

**Results:**

TFDG was found to inhibit proinflammatory factors (IL-6, TNF-*α*, iNOS, and PGE) and matrix-degrading enzymes (MMP13, MMP3, and ADAMTS5) expression and protected extracellular matrix components of chondrocytes (ACAN, COL2, and SOX9). TFDG accelerated the scavenging of ROS caused by IL-1*β* according to the Nrf2 signaling pathway activation. At the same time, TFDG suppressed the PI3K/AKT/NF-*κ*B and MAPK signaling pathways to delay the inflammatory process. The cartilage of DMM rats receiving TFDG showed lower Osteoarthritis Research Society International (OARSI) scores and expressed higher levels of COL2 and Nrf2 compared with those of rats in the DMM group.

**Conclusion:**

TFDG could protect cartilage from degradation and alleviate osteoarthritis in rats, which suggests that TFDG has potential as a drug candidate for OA therapy.

## 1. Introduction

A chronic degenerative disease of total joints, osteoarthritis (OA) affects most easily the knee joint, and in second instance the hand and hip joints [1]. Its clinical symptoms include joint swelling and pain, joint stiffness and limited movement, atrophy of the muscles around the joint, and frictional sounds during movement, which eventually leads to joint deformity in the late stage of the disease [2]. There are many risk factors for OA, including age, obesity, being female, and history of knee joint injury; among these, age is the most relevant [3, 4]. Therefore, along with the increasing incidence of obesity due to population aging and dietary changes, the global prevalence of OA is also increasing. It is estimated that 242 million people are affected by OA worldwide [5]. The high incidence of OA, combined with the irreversibility of disease progression, ranks it alongside diabetes as one of the fastest-growing causes of disability worldwide; in particular, OA is expected to become the fourth leading cause of disability by 2020 [6–8]. At present, because of its pathogenesis, OA is difficult to cure. Therefore, the corresponding treatment plan is provided according to the situation of the patient and disease stage. In general, for early lesions, OA patients with no obvious symptoms usually adopt preventive nondrug treatment [9]. Furthermore, loss of weight, avoidance of daily activities that exert pressure on the joints for a long time, moderate movement to enhance joint stability, and physical therapy can help delay the progression of OA as much as possible [10]. Conversely, OA patients with obvious clinical symptoms may require nondrug therapy, nonsteroidal anti-inflammatory drugs, analgesic drugs, antianxiety drugs, or proprietary Chinese medicine to relieve pain caused by OA [11, 12]. In particular, intra-articular injection of the drug will relieve pain more quickly and effectively. However, conventional drugs and physical therapy are no longer effective when the disease is in its final stages, and surgery is the only option to relieve pain and correct deformities [13]. Surgery practices mainly consist in articular cartilage repair, arthroscopic debridement, osteotomy, and joint replacement. The latter is currently the most widely used method worldwide, but its postoperative complications are still difficult to resolve completely [14, 15]. Therefore, it is urgent to deepen current knowledge of OA pathogenesis in order to find better therapeutic options for this disease.

Previous studies have explored the pathogenesis of OA, but the exact mechanism remains unclear. A large number of cytokines and signaling pathways play a synergistic role in the pathogenesis of OA, and the related inflammatory responses and oxidative stress have been widely studied [16, 17]. Cytokines that trigger inflammatory responses, such as interleukin 1 (IL-1) and tumor necrosis factor (TNF), are the most crucial molecules supporting OA pathogenesis [18]. Previous clinical studies have found the synovial fluid of OA patients have escalated IL-1*β* secretion [19]. Moreover, Tesche and Miosge reported IL-1*β* overexpression in osteoarthritic cartilage [20]. These findings implied that IL-1*β* plays a relevant role in OA. Inflammatory factors such as IL-1*β* can either directly promote the synthesis of matrix-degrading enzymes in chondrocytes, thereby promoting extracellular matrix degradation, or amplify the inflammatory response and accelerate chondrocytes apoptosis by activating downstream signaling pathways or other inflammatory factors [21, 22]. For example, IL-1*β* can activate the ERK, p38, and JNK, NF-*κ*B, and PI3K pathways or other cytokines to accelerate the progression of arthritis [23–25]. Oxidative stress also plays a major role in OA progression [26]. In chondrocytes, mitochondrial electron transfer generated mostly reactive oxygen species (ROS). Upon normal mitochondrial function, the production and clearance of ROS are maintained in a dynamic balance [27]. However, when mitochondria are dysfunctional and ROS generation exceeds the mitochondrial clearance capacity, such balance is disrupted, and excessive ROS accumulation lead to cell damage [28, 29]. Excess ROS, when released in chondrocytes, damage DNA and proteins, induce mitochondrial dysfunction, and activate proinflammatory mediators and matrix-degrading enzymes. Conversely, the application of antioxidants can significantly improve the condition of chondrocytes [30]. In summary, both inflammation and oxidative stress are involved in the pathogenesis of OA and can mutually reinforce. Therefore, inhibiting inflammation and oxidative stress is believed to be an effective approach to treat OA.

Many natural active substances in herbs and plants have been proven to exert beneficial biological effects as they can play anti-inflammatory and antioxidant roles both *in vitro* and *in vivo* [31]. Therefore, further research on these substances may yield new antiarthritic drugs. In particular, theaflavin-3-3′-digallate (TFDG), the active substances extracted from black tea, have been shown to exert a variety of biological effects. For instance, TFDG have been reported to alleviate cardiomyocyte reperfusion through the inhibition of autophagy [32]. These molecules can also improve lipopolysaccharide-induced bronchitis in rats by suppressing the release of inflammatory factors [33]. As for the skeletal system, previous studies have found that theaflavins can inhibit the activation of osteoclasts and prevent bone loss in castrated mice, thereby preventing osteoporosis [34]. Finally, Liu and Li reported that TFDG alleviates rheumatoid arthritis by inhibiting the NF-*κ*B and MAPK pathways in mice model [35]. Therefore, we aimed to investigate whether TFDG can bring into play anti-inflammatory and antioxidant function in OA models, verify whether TFDG treatment can retard OA progression, and further study its mechanism of action.

## 2. Materials and Methods

### 2.1. Regents and Antibodies

TFDG (CSN20910) was obtained from CNS Pharmaceuticals (Chicago, IL, USA), configured with ethyl alcohol (Sigma-Aldrich, USA), and diluted to a concentration of 10 mM. Recombinant IL-1*β* of rats was offered by PeproTech Inc. (East Windsor, NJ, USA) and dissolved in a 5% trehalose solution. Primary antibodies against collagen II (ab188570), COX-2 (ab179800), MMP3 (ab52915), ADAMTS5 (ab41037), SOX9 (ab185230), iNOS (ab178945), and SOD2 (ab68155) were acquired from Abcam (Cambridge, UK). Antibodies against p85 (#4257), p110 (#4255), AKT (#9272), phospho-AKT (#4060), ERK1/2 (#9102), phospho-JNK (#4668), p38 (#8690), phospho-p38 (#4511), phospho-ERK1/2(#4370), phospho-p65 (#3033), p65 (#8242), phospho-I*κ*B*α* (#2859), I*κ*B*α* (#9242), and JNK (#9252) were supplied by CST (Beverly, USA). Anti-Nrf2 (16396-1-AP), anti-ACAN (13880-1-AP), anti-MMP13 (18165-1-AP), anti-*β*-actin (20536-1-AP), and anti-HO-1 (10701-1-AP) antibodies were taken from Proteintech (Wuhan, China).

### 2.2. Extraction and Culture of Rat Chondrocytes

6–8-week-old Sprague-Dawley (SD) rats supplied by the Experimental Animal Center of Soochow University were used for primary chondrocytes isolation. SD rats were sacrificed and then confirmed dead according to their lack of respiration and heartbeat. The articular cartilages were exposed and separated from their subchondral bone. The cartilage was chopped into little pieces, placed in cell culture dishes, and digested using type II collagenase (0.2%, Thermo Fisher Scientific) for 6 h at 37°C. DMEM/F12 containing 100 U antibiotics and 10% fetal bovine serum (FBS) (Thermo Fisher Scientific) were used for collected chondrocytes culturing at 37°C and 5% CO_2_. The medium was replaced every 3 days and chondrocytes were passaged with 0.25% trypsin/ethylenediaminetetraacetate (Thermo Fisher) at 90% cell density. Chondrocytes from the first and second passages were used for study.

### 2.3. Cell Proliferation Analysis

The effects of TFDG on cell viability were monitored by CCK-8 KIT (Beyotime, Shanghai, China). Chondrocytes in 96-well plates (5000 cells/well) were cocultrued with multiple concentration of TFDG after adhering to the wells; untreated cells were set for negative control. After discarding the original culture medium and washing the wells twice, the chondrocytes were handled with CCK-8 solution (10%) at 37°C for 1 h. Microplate reader (BioTek, USA) was used for measuring the optical density (OD) of the cell cultures at 450 nm. The cell viability was calculated according to the following formula. Cell viability = [(OD_experimental well_ − OD_blank well_)/(OD_control well_ − OD_blank well_)]∗100%.

### 2.4. Detection of Intracellular Reactive Oxygen Species

After treatment with IL-1*β* and TFDG for 24 h, chondrocytes cultured in six-well plates were collected and cocultured with 10 *μ*M 2′,7′-dichlorofluorescein diacetate (DCFH-DA) at 37°C for 20 min. The mean fluorescence intensity was measured by flow cytometry (Thermo Fisher Scientific), and FlowJo software 10.7 (BD Life Sciences, Franklin Lakes, NJ, USA) was employed for dealing with data.

### 2.5. RNA Isolation and Quantitative Real-Time PCR

TRIzol® (Thermo Fisher Scientific) was applied for total RNA extracting from chondrocytes, and RNA reverse transcription was performed using the PrimeScript RT Reagent Kit (Takara, Tokyo, Japan) to obtain complementary DNA (cDNA). Real-time reverse transcription quantitative PCR (RT-qPCR) was carried out on a Real-Time PCR System (Bio-Rad, USA) using iTaq™ Universal SYBR® Green Super Mix with 1 *μ*g of cDNA and primers. *Gapdh* as an internal reference was used to normalize gene expression, which was calculated using the 2^−(*ΔΔ*Ct)^ method. Supplementary Table 1 shows the primer sequences used to detect target genes.

### 2.6. Western Blotting

Chondrocytes cultured in six-well plates were washed twice, and RIPA lysis buffer was utilized for total protein isolation. Nuclear and Cytoplasmic Protein Extraction Kit (Beyotime) was used for separating proteins from nuclei and cytoplasm. Quantified protein concentration was detected using a Pierce™ BCA Kit (Thermo Fisher Scientific); equal proteins diluted in loading buffer were isolated by 10% SDS-PAGE and then delivered to nitrocellulose membranes. Western blocking buffer (Beyotime) was utilized for membranes blocking which then was incubated with the corresponding primary antibodies overnight at 4°C. Next, at room temperature, incubation with horseradish peroxidase- (HRP-) conjugated secondary antibodies was performed for 1 h. Membranes were exposed using ECL (Thermo Fisher Scientific) and visualized using a GS-800 scanner (Bio-Rad). ImageJ software (National Institutes of Health, Bethesda, MD, USA) was used for analyzing the density of band.

### 2.7. Immunofluorescence

24-well plates were applied for culture of chondrocytes treated with IL-1*β* or TFDG. After cells reaching 50% density, the medium was replaced with PBS for rinsing cells twice, subsequently experienced fixation for 4% paraformaldehyde for 10 min, and then 0.25% Triton X-100 was used for cells permeabilization for 15 min and then blocked for 1 h. Cells were then soaked into diluted antibodies solution at 4°C overnight, and at room temperature, cells were incubated with fluorescent secondary antibodies for 1 h. 4′,6-Diamidino-2-phenylindole (DAPI, Abcam) was used to counterstain nuclei via incubation for 10 min. Fluorescence microscope (Zeiss, Germany) was used for obtaining pictures.

### 2.8. siRNA Transfection

After reaching 50% confluence, chondrocytes cultured in six-well plates were transfected with siNrf2 and siNC, which were provided by RiboBio (Guangzhou, China), using Lipofectamine 3000 transfection reagent on the base of the instruction for 6 h at 37°C. Transfected cells were used for subsequent experiments.

### 2.9. Animal Surgical Model and Treatments

All animal experiments were checked by the Ethics Committee for Animal Experiments at the First Affiliated Hospital of Soochow University. Thirty 6–8-week-old SD rats provided by the Experimental Animal Center of Soochow University were randomly assigned to three groups: the sham, OA, and OA+TFDG. A knee OA model was established according to giving rise to surgical destabilization of the medial meniscus (DMM). After all rats were anesthetized inhaling 2% isoflurane, a medial parapatellar incision in the right knee joint was performed to disclose the medial meniscotibial ligaments (MML). Rats in the OA and TFDG-injected groups underwent excision of MML, while the sham group retained intact MML. Intra-articular injections were performed 2 weeks after modeling. 100 *μ*L of this saline solution, containing 4 mM TFDG, was infused into the right knee joint of rats in the TFDG group every 2 days for 6 weeks. Rats in other two groups received the same volume of saline solution in the knee joint.

### 2.10. Histology and Immunohistochemistry

All groups of rats raised for 8 weeks were sacrificed for collecting the right knee joints and 4% paraformaldehyde-fixed tissue underwent decalcification in 10% EDTA for 1 month. The specimens wrapped in wax were cut into 6 *μ*m thick slices for hematoxylin-eosin (HE) and safranin O/fast green (SO) staining, and the severity of cartilage degradation was estimated according to the Osteoarthritis Research Society International (OARSI) scoring system. Immunohistochemistry was performed as follows: xylene and gradient alcohol were used for dewaxing and dehydrating slices. After removal of endogenous peroxidase, the slices were incubated with 0.25% tyrisin for 60 min to restore the masked epitope, and then, goat serum was used for blocking slices for 20 min. Slices were then covered with anti-nrf2 and anti-COLII antibodies overnight at 4°C, and after that, secondary antibodies were used for 1 h at room temperature. Finally, a diaminobenzidine (DAB) substrate kit was used to visualize the antigens, and hematoxylin was used for nuclear counterstaining. The calculation method of the staining ratio of positive cells is the number of stained cells/total number of cells in limited area, and the number of positive cells was marked and analyzed using ImageJ. All images were photoed under a Zeiss Axiovert 40CFL microscope.

### 2.11. Enzyme-Linked Immunosorbent Assay (ELISA)

The medium of cells treated with various concentrations of TFDG was collected and added to appropriate 96-well plates included in the ELISA kit (MultiSciences, China). TNF-*α*, PEG2, and IL-1*β* cytokine concentrations in the medium were quantified according to the detection of stop buffer absorbance at 450 nm and calculated through the standard curve.

### 2.12. Statistical Analysis

GraphPad Prism 8.0 software (San Diego, CA, USA) was used for performing all data processing. One-way analysis of variance (ANOVA) and Tukey's test were used for multiple comparisons. Comparison between two groups was evaluated using Student's *t*-test. All experiments were performed at least three times, and *p* < 0.05 was regarded as statistical significance.

## 3. Results

### 3.1. Effect of TFDG on Cell Viability

**Figure 1:**
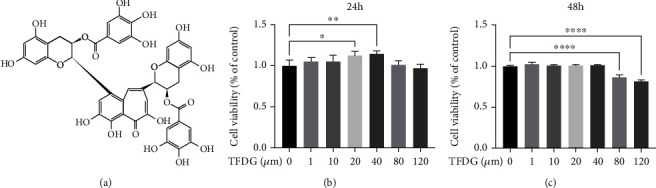
The structure of TFDG and effect of TFDG on cell viability. (a) The molecular structure of TFDG. (b, c) Different concentrations of TFDG (1, 10, 20, 40, 80, or 120 *μ*M) to determine its cytotoxicity at 24 h and 48 h. The bar graph shows the mean ± SD of data (*n* = 6). ^∗^*p* < .05, ^∗∗^*p* < .01, ^∗∗∗^*p* < .001, and ^∗∗∗∗^*p* < .0001.

The molecular structure of TFDG is shown in Figure 1(a). Various concentrations of TFDG (1, 10, 20, 40, 80, or 120 *μ*M) were added to the cell culture medium to determine its cytotoxicity. Chondrocytes treated with TFDG at concentrations lower than 80 *μ*M showed increased viability with respect to control cells; in particular, treatment with 20 *μ*M and 40 *μ*M TFDG significantly promoted cell proliferation at 24 h (*p* < 0.05), although this effect was not apparent at 48 h. When the concentration of TFDG exceeded 80 *μ*M, its toxicity was not obvious at 24 h, but it significantly inhibited cell proliferation at 48 h (Figures 1(b) and 1(c)). Based on the above results, TFDG concentrations of 20 *μ*M and 40 *μ*M were used in the following experiments.

### 3.2. TFDG Alleviates Inflammatory Responses in Chondrocytes

**Figure 2:**
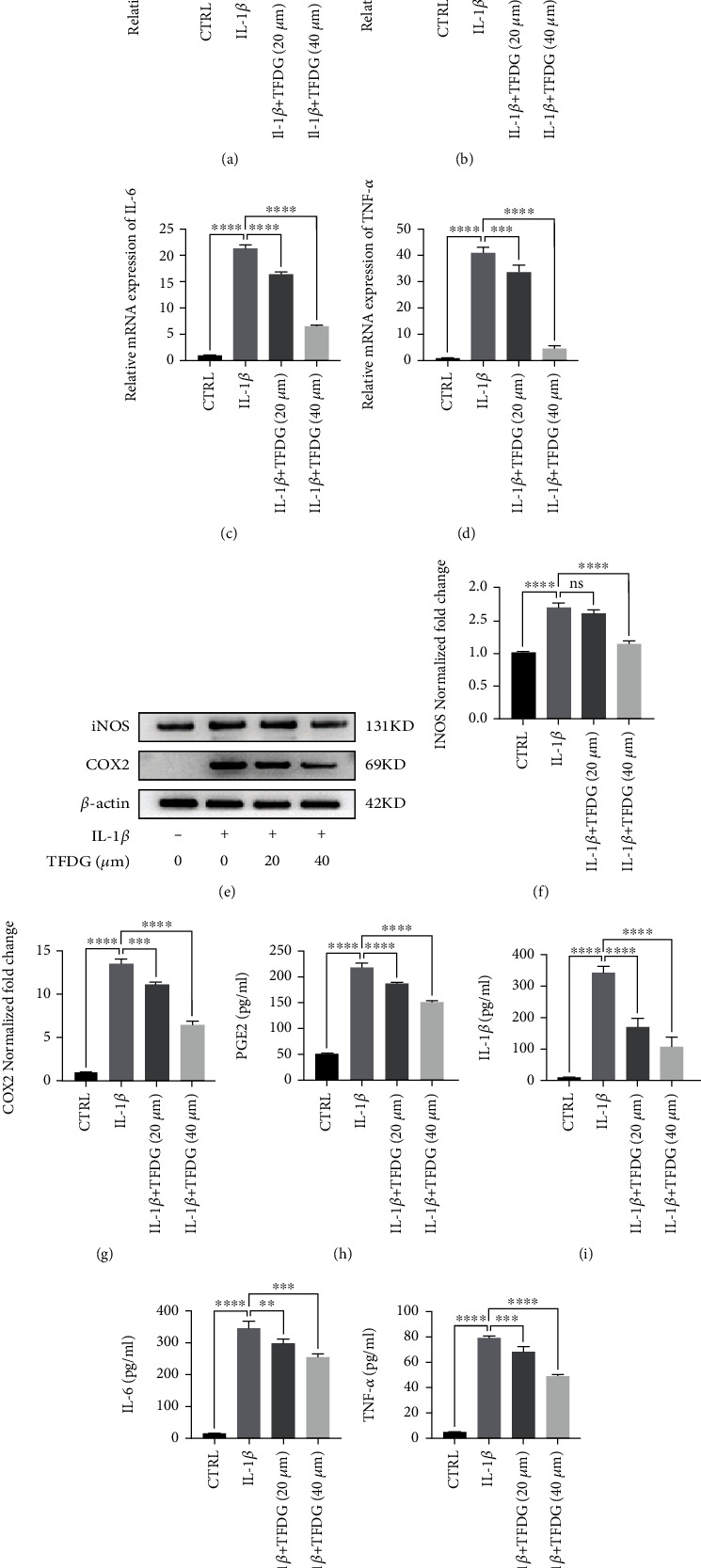
TFDG alleviates inflammatory responses in chondrocytes. (a–d) The transcription levels of iNOS, Ptgs2, TNF-*α*, and IL-6 were determined by RT-qPCR. (e–g) The protein expressions of iNOS and COX2 were analyzed by western blotting. The grey values normalized with *β*-actin and control group were quantified by ImageJ. (h–k) Proinflammatory cytokines such as PGE2, IL-1*β*, IL-6, and TNF-*α* from chondrocytes in different groups detected by ELISA. The bar graph shows the mean ± SD of data (*n* = 4). ^∗^*p* < .05, ^∗∗^*p* < .01, ^∗∗∗^*p* < .001, and ^∗∗∗∗^*p* < .0001.

IL-1*β* (10 ng/mL) was used to simulate the environment where chondrocytes were affected by inflammation. Compared to the control group, the transcription levels of *iNOS*, *ptgs2*, *Tnf-α*, and *Il-6* were significantly upregulated in chondrocytes treated with IL-1*β* which also led to elevating iNOS and COX-2 protein expression. However, TFDG reversed the effect of IL-1*β* on chondrocytes as it downregulated the expression of the above-mentioned inflammation-related factors at both the gene and protein expression (Figures 2(a)–2(d)). ELISA results also suggested that TFDG inhibits IL-1*β*-induced secretion of IL-1*β*, PGE2, IL-6, and TNF-*α* from chondrocytes (Figures 2(h)–2(k)).

### 3.3. TFDG Protects the Cartilage Matrix of Chondrocytes

**Figure 3:**
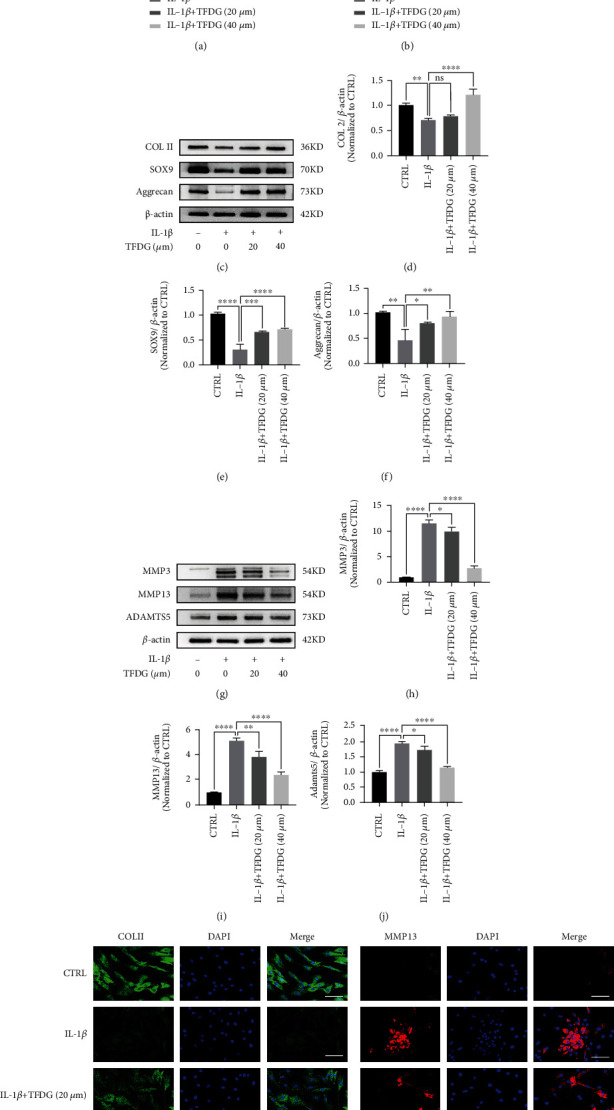
TFDG protects the cartilage matrix of chondrocytes. (a and b) The gene levels of *Mmp3*, *Mmp13*, and *Adamts5* and *Col2a1*, *Sox9*, and *ACAN* were analyzed by RT-qPCR. (c–j) The protein expressions of COL2, SOX9, aggrecan, MMP13, MMP3, and ADAMTS5 were analyzed by western blotting. The grey values normalized with *β*-actin and control group were quantified by ImageJ. The bar graph shows the mean ± SD of data (*n* = 4). ^∗^*p* < .05, ^∗∗^*p* < .01, ^∗∗∗^*p* < .001, and ^∗∗∗∗^*p* < .0001. (k and l) COL2 and MMP13 protein expressions were detected by immunofluorescence. Scale bar: 100 *μ*m.

IL-1*β* treatment suppressed *Col2a1*, *Sox9*, and *ACAN* expressions in chondrocytes, while treatment with TFDG partly restored the expression of these genes associated with matrix synthesis (Figure 3(a)). At the same time, RT-qPCR revealed that IL-1*β* remarkably promoted the transcription of *Adamts5*, *Mmp3*, and *Mmp13* which was prevented by TFDG treatment (Figure 3(b)). Western blotting results also confirmed that IL-1*β* dramatically downregulated COLII, SOX9, and aggrecan expression while upregulating matrix-degrading enzymes expression, including MMP3, MMP13, and ADAMTS5 at the protein level; however, TFDG blocked this effect (Figures 3(c)–3(j)). Consistently, an immunofluorescence assay confirmed that TFDG promoted COLII synthesis but suppressed IL-1*β*-induced MMP13 expression (Figures 3(k) and 3(l)).

### 3.4. TFDG Reduces IL-1*β*-Induced ROS Accumulation by Activating the Nrf2 Pathway

**Figure 4:**
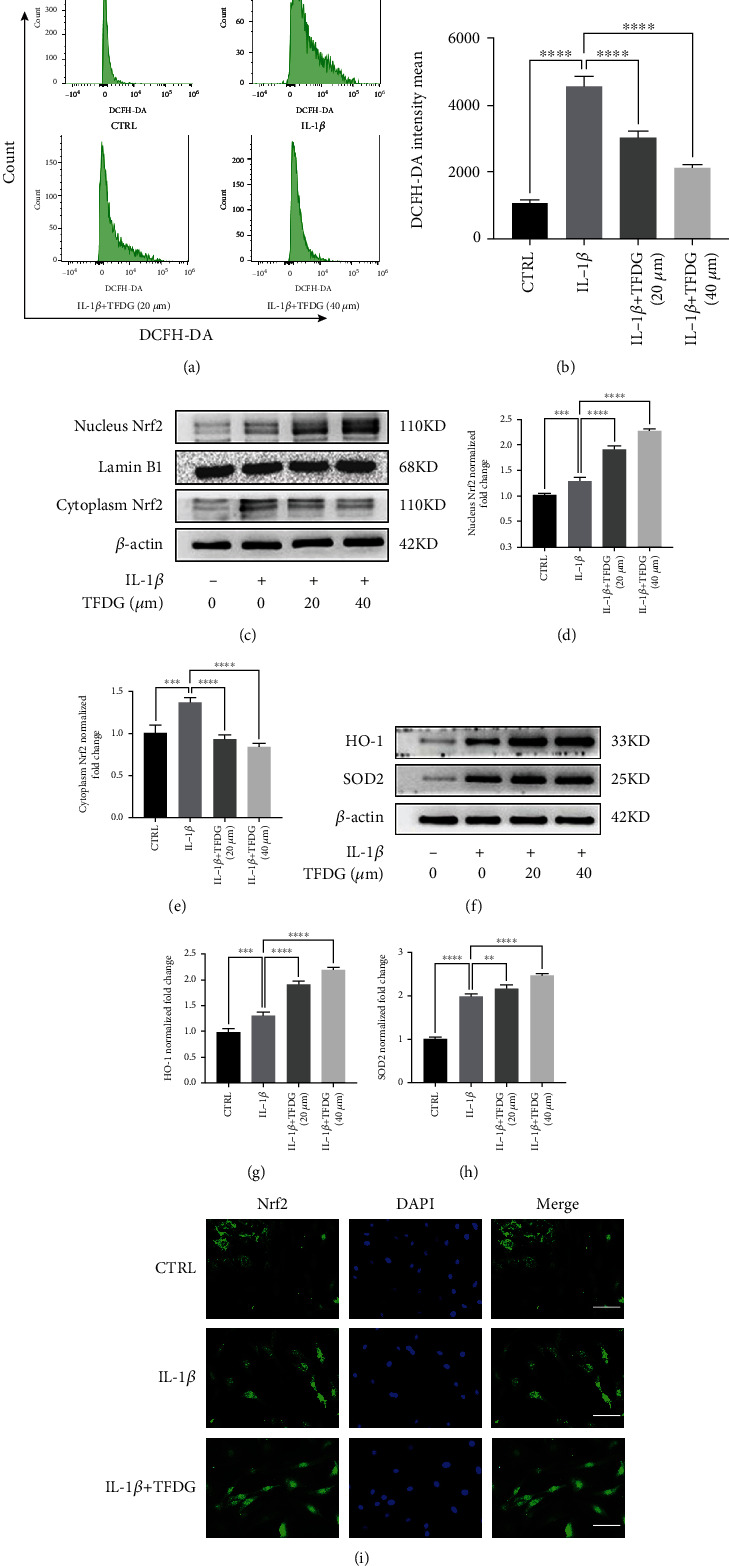
TFDG reduces IL-1*β*-induced ROS accumulation by activating the Nrf2 pathway. (a and b) The intensity mean of ROS marked by DCFH-DA was quantified by flow cytometry. (c–h) The protein expressions of Nrf2 in nucleus and cytoplasm, HO-1, and SOD2 were analyzed by western blotting. The grey values of Nrf2 protein in nucleus normalized with Lamin B and other protein normalized with *β*-actin and control group were quantified by ImageJ. The bar graph shows the mean ± SD of data (*n* = 4). ^∗^*p* < .05, ^∗∗^*p* < .01, ^∗∗∗^*p* < .001, and ^∗∗∗∗^*p* < .0001. (i) Nrf2 protein expressions were detected by immunofluorescence. Scale bar: 100 *μ*m.

The production of intracellular ROS stimulated by IL-1*β* was measured by inspecting changes in DCFH-DA fluorescence. After being treated with IL-1*β* and/or TFDG, chondrocytes were collected and incubated with DCFH-DA. Subsequently, DCFH-DA fluorescence was measured by flow cytometry, and stronger green fluorescence intensity indicated greater ROS accumulation in chondrocytes. Flow cytometric analysis suggested that IL-1*β* treatment resulted in a robust production of ROS in cells, whereas TFDG treatment assisted chondrocytes in scavenging ROS and reduced their accumulation (Figures 4(a) and 4(b)). To determine the mechanism by which TFDG inhibits ROS accumulation, the activity status of the Nrf2/HO-1 signaling pathway, which plays an important role in ROS detoxification, was further investigated. Western blotting results demonstrated that the Nrf2/HO-1 signaling pathway is involved in the ROS-scavenging activity of TFDG. In fact, TFDG significantly promoted the expression of Nrf2 in the nucleus, where it binds to ARE elements, as well as HO-1 and SOD2 expression. Conversely, IL-1*β* upregulated Nrf2 expression mainly in the cytoplasm; moreover, HO-1 and SOD2 expressions were upregulated only slightly compared to that of the control (Figures 4(c)–4(h)). Similarly, immunofluorescence also revealed that Nrf2 mainly accumulated in the nuclei, confirming that TFDG can enhance Nrf2 translocation into the nucleus (Figure 4(i)).

### 3.5. The Blockade of Nrf2/HO-1 Signaling Partly Reverses the Protection of TFDG

**Figure 5:**
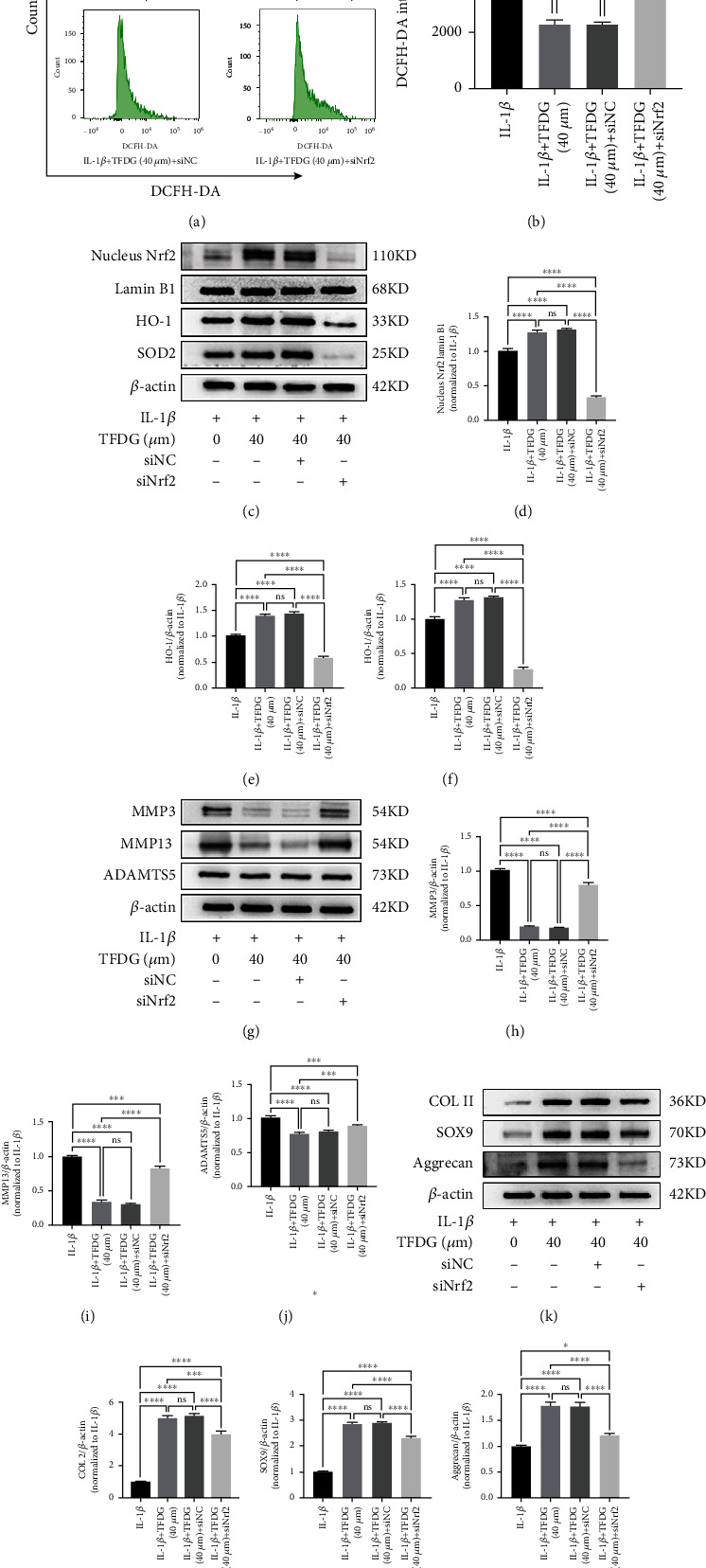
The blockade of Nrf2/HO-1 signaling partly reverses the protective effect of TFDG. (a and b) The intensity mean of ROS marked by DCFH-DA was quantified by flow cytometry. (c–n) The protein expressions of Nrf2 in nucleus, HO-1, SOD2, COL2, SOX9, aggrecan, MMP13, MMP3, and ADAMTS5 were analyzed by western blotting. The grey values of Nrf2 protein in nucleus normalized with Lamin B and other protein normalized with *β*-actin and control group were quantified by ImageJ. The bar graph shows the mean ± SD of data (*n* = 4). ^∗^*p* < .05, ^∗∗^*p* < .01, ^∗∗∗^*p* < .001, and ^∗∗∗∗^*p* < .0001.

To verify that the Nrf2/HO-1 signaling pathway participates in the protection against OA offered by TFDG, chondrocytes were transfected with siNrf2 to knock down *Nrf2* expression; siNC was used as a negative control to account for the effects of the transfection process. According to flow cytometric analysis, green fluorescence intensity remained elevated in siNrf2-transfected cells, indicating that the inhibition of the Nrf2 weakened the clearance effect of TFDG on ROS and thus led to ROS accumulation (Figures 5(a) and 5(b)). Western blot analysis indicated that Nrf2 expression in the nucleus decreased significantly upon siNrf2; in turn, this suppression also resulted in HO-1 and SOD2 protein expression decreasing (Figures 5(c)–5(f)). The blockade of the Nrf2/HO-1 pathway also interfered with the defendant of the cartilage matrix caused by TFDG. In fact, compared to those of the TFDG group and the TFDG+siNC group, the expression of COL2A1, SOX9, and aggrecan in chondrocytes was decreased in the TFDG+siNrf2 group, although it remained higher than that of the IL-1*β* group. The opposite was observed for MMP3, MMP13, and ADAMTS5 protein expressions in the TFDG+siNrf2 group, which was higher than that of the TFDG group and the TFDG+siNC group but lower than that of the IL-1*β* group (Figures 5(g)–5(n)).

### 3.6. TFDG Inhibits Inflammation-Related Pathways Induced by IL-1*β*

**Figure 6:**
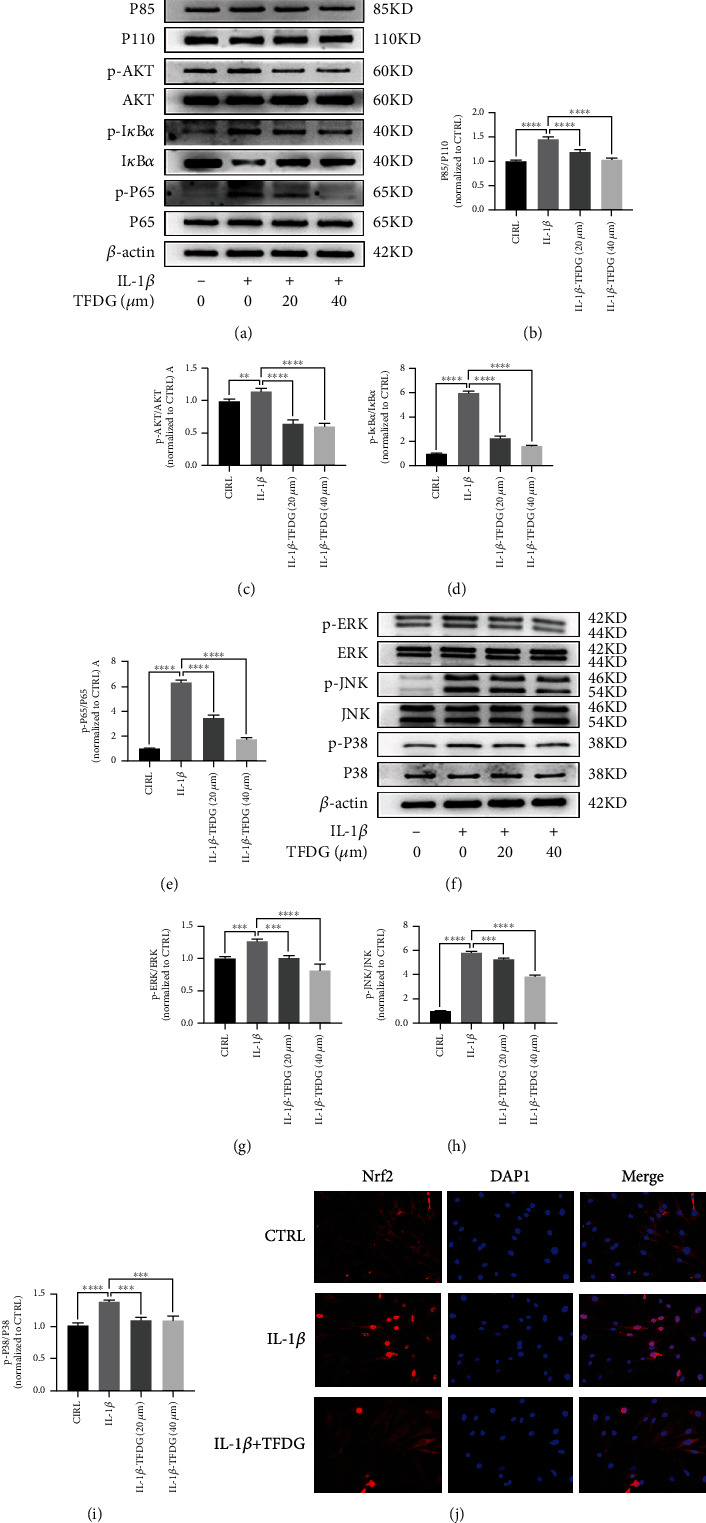
TFDG inhibits inflammation-related pathways induced by IL-1*β*. (a–i) The protein expressions of phosphorylated PI3K, AKT, p65, I*κ*B*α*, ERK, JNK, and p38 were analyzed by western blotting. The grey values normalized with control group were quantified by ImageJ. The bar graph shows the mean ± SD of data (*n* = 4). ^∗^*p* < .05, ^∗∗^*p* < .01, ^∗∗∗^*p* < .001, and ^∗∗∗∗^*p* < .0001. (j) P65 protein expressions were detected by immunofluorescence. Scale bar: 100 *μ*m.

The MAPK and PI3K/AKT/NF-*κ*B signaling pathways are believed to hasten OA, and some article showed that TFDG can inhibit these pathways in other diseases. To confirm that TFDG can repress these two pathways, western blotting assays were carried out. In IL-1*β*-treated chondrocytes, the expression of phosphorylated PI3K, AKT, p65, I*κ*B*α*, ERK, JNK, and p38 was significantly upregulated, whereas I*κ*B*α* expression was downregulated, which represents the activation of the inflammation-related axis. Nevertheless, the expression of phosphorylated PI3K, AKT, p65, I*κ*B*α*, ERK, JNK, and p38 was reduced, while I*κ*B*α* expression was increased in the presence of TFDG, which hindered the activation of these pathways (Figures 6(a)–6(i)). An immunofluorescence assay was performed to observe changes in p65 expression and localization: the results demonstrated that the in control group p65 protein was mostly in the cytoplasm, while IL-1*β* treatment caused p65 transfer into the nucleus. However, in TFDG-treated group, p65 expression restored in the cytoplasm (Figure 6(j)).

### 3.7. TFDG Prevented DMM-Induced Cartilage from Degradation

**Figure 7:**
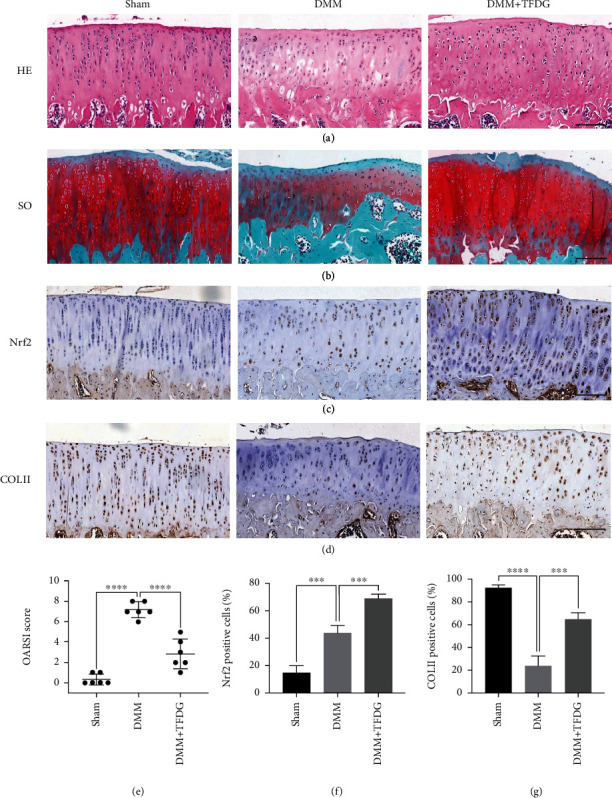
TFDG protects DMM-induced cartilage from degradation. (a and b) The rats of joint sections were stained by HE and safranin O/fast green. (c and d) The COLII and Nrf2 protein expressions in cartilage were determined by immunohistochemistry staining. (e) The OARSI scores of cartilages in different groups. (f and g) The Nrf2 and COLII positive cells were quantified. The bar graph shows the mean ± SD of data (*n* = 4). ^∗^*p* < .05, ^∗∗^*p* < .01, ^∗∗∗^*p* < .001, and ^∗∗∗∗^*p* < .0001. Scale bar: 100 *μ*m.

To explore the effects of TFDG *in vivo*, rats in the OA and TFDG-injected groups underwent DMM surgery accompanied or not by TFDG treatment. All rats were sacrificed 8 weeks after surgery, and the extracted knee joints were sliced into sections. To analyze the degree of cartilage destruction, the sections were stained by HE and safranin O/fast green. The articular surface from the control group was complete, and the cartilage could secrete abundant glycosaminoglycans. However, OA induced severe joint wear and loss of glycosaminoglycans in the cartilage. Conversely, the cartilage of rats that had received TFDG injection for 6 weeks after surgery was protected from degradation and could accumulate more glycosaminoglycans (Figures 7(a) and 7(b)). The severity of OA was quantified according to the OARSI scoring system: rats in the TFDG group exhibited higher scores than those in the OA group (Figure 7(e)). Immunohistochemistry assays revealed a higher number of cells positive for COLII and Nrf2 expression in the TFDG-injected group than in the OA group; this suggests that TFDG can protect the cartilage matrix from inflammation via activation of Nrf2 (Figures 7(c), 7(d), 7(f), and 7(g)).

## 4. Discussion

According to the literature, the occurrence of OA depends on a variety of mechanisms. In addition to inflammatory responses, mechanical load, oxidative stress, and cell senescence may all promote the degradation and inhibit the synthesis of the extracellular matrix, resulting in cartilage damage and ultimately OA [36–39]. Therefore, early suppression of inflammatory responses in chondrocytes and mitigation of oxidative stress levels may delay the onset of OA. Many natural compounds with antioxidant and anti-inflammatory properties have been explored as candidates for delaying the progression of OA [40]. TFDG is a characteristic compound of black tea, and its excellent anti-inflammatory and antioxidant properties have been demonstrated in a variety of diseases [41]. For example, Ai et al. found that TFDG treatment could reduce RANKL-induced intracellular ROS accumulation in RAW cells and inhibit osteoclast formation, thereby promoting osteogenesis and alleviating bone loss in immunized mice [34]. Similarly, San Cheang et al. found that TFDG can delay hypertension-induced apoptosis of endothelial cells through their antioxidant properties [41]. Therefore, we aimed to investigate whether TFDG can exert anti-inflammatory and antioxidant effects in chondrocytes to delay the progression of arthritis. To determine the appropriate dose of TFDG for chondrocytes in an inflammatory environment *in vitro*, we selected six gradually increasing concentrations (1, 10, 20, 40, 80, and 120 *μ*M) by referring to the application of TFDG in other diseases. Then, the CCK-8 assay was used to measure the effect of TFDG at these six concentrations on chondrocyte proliferation, to facilitate the selection of a low concentration and a high concentration for subsequent experiments. According to the results of the CCK-8 assay, TFDG is safe for chondrocytes when administered at a concentration below 40 *μ*M but can significantly inhibit the growth of chondrocytes after treatment at a higher dose. At the same time, TFDG was found to promote the growth of chondrocytes within 24 h, especially at a concentration of 40 *μ*M, but this effect was no longer obvious after 48 h. Therefore, the treatment duration was 24 h, and 20 *μ*M TFDG was used for the low-concentration treatment group, while 40 *μ*M TFDG was used for the high-concentration treatment group. After determining the working dose of TFDG, we treated chondrocytes with IL-1*β* and/or TFDG and observed changes in inflammatory indexes, matrix synthesis, and degradation indexes. We found that IL-1*β*-mediated inhibition of chondrocyte proliferation was alleviated by combined IL-1*β*/TFDG treatment. TFDG was found to exert a dose-dependent effect on decreasing inflammatory indicators in chondrocytes and to significantly downregulate IL-1*β* and TNF-*α* expression; similarly, the expression levels of COX-2 and iNOS, two landmark inflammatory proteins, were significantly reduced, especially in the high-concentration TFDG-treated group; all other indicators were close to normal levels. When measuring matrix synthesis and degradation indexes, we found that the expression of type II collagen and proteoglycans at both the gene and protein levels increased with increasing TFDG concentrations, while the expression of *Mmp3*, *Mmp13*, and *Adamts5* decreased with increasing TFDG concentrations.

Previous studies have found that oxidative stress promotes the onset of OA and plays a key role in its progression [42, 43]. On the other hand, TFDG has been reported to be an effective antioxidant. Consistent with this background, the results of our flow cytometry assay using the fluorescent probe DCFH-DA, a marker of ROS in cartilage cells, aiming to quantify the indirect fluorescence intensity of intracellular ROS, showed that IL-1*β* can induce the production of intracellular ROS, but TFDG treatment can counteract such accumulation. Keap1/Nrf2, as a classical antioxidant pathway, is expressed in multiple organs where it plays an important role [44]. Under normal conditions, the DGR region of Keap1 binds to the DLG and ETGE sequences of Nrf2, which stabilizes Nrf2 in the cytoplasm and induces Nrf2 degradation through the ubiquitination system to inhibit its activity [45]. Keap1 transforms its connection with Nrf2 by sensing changes in electrons and ROS which reduces the degradation of Nrf2 through ubiquitination, accelerating the accumulation of nucleus Nrf2 binding with the promoter ARE to promote transcription and translation of downstream antioxidant proteases. Therefore, the imbalance or deletion of Keap1/Nrf2 pathway often leads to the damage of the organ and the progression of the corresponding disease [46]. On the contrary, upregulating keap1/Nrf2 expression can effectively resist chronic inflammation, degenerative changes, and even cancer caused by oxidative stress [47–49]. Adeel Safdar found that keap1/Nrf2 pathway is also involved in the musculoskeletal diseases. The ROS generation rate and Nrf2 expression level are both low in young individuals. However, with the aging of individuals, at first, the increased Nrf2 expression is consistent with the increased ROS level. Thus, cells can still maintain oxidative stress balance, however, which is transient. With aging, transcription of Nrf2 regulated by ARE will be destroyed eventually, resulting in the decrease of Nrf2 expression, and the accumulation of ROS is difficult to clear, leading to cell apoptosis and musculoskeletal degeneration [50]. Cai et al. found that after the onset of OA in a murine model of DMM, the expression of Nrf2 and its downstream antioxidant enzymes significantly increased to limit OA progression, while Nrf2-KO mice lost the ability to activate Nrf2 to reduce MMPs and protect cartilage [51]. Therefore, the spontaneous increase of Nrf2 expression in the aged population after the occurrence of OA is insufficient to remove the intracellular ROS, and further stimulation of Nrf2 expression through exogenous intervention may be an effective means to treat OA and protect cartilage. Our study found that after the chondrocytes were stimulated by IL-1*β*, the expressions of Nrf2 in the nucleus and the downstream antioxidant proteases SOD2 and HO-1 were slightly increased, so the intracellular ROS still maintained a high level. After TFDG intervention, the expression of Nrf2 in the nucleus, SOD2, and HO-1 was significantly increased, and the scavenging of ROS was accelerated. To confirm that the activation of Nrf2 pathway is an important way for TFDG to protect articular cartilage, we used siRNA to interfere with Nrf2 transcription and found that the effect of TFDG was reversed and the levels of Nrf2 in the nucleus as well as SOD2 and HO-1 in the cytoplasm decreased, while intracellular ROS increased again. These results indicated that TFDG reduced ROS in the articular chondrocytes relying on Nrf2 mediated and the protective effect of TFDG on the extracellular matrix of chondrocytes was also weakened. In vivo immunohistochemical results also showed Nrf2 activation in OA group, but Nrf2 activation was more significant after TFDG injection, which was conducive to maintaining the homeostasis of the joint environment.

Previous studies found that after Nrf2/HO-1 pathway was inhibited, the protective effect of TFDG on chondrocytes was not completely lost, which may depend on the anti-inflammatory effect of TFDG. Therefore, PI3K/AKT/NF-*κ*B and MAPK pathways were investigated successively. The NF-*κ*B signaling pathway is responsible for the transmission and amplification of inflammatory signals and activated NF-*κ*B was seen in the synovium of both osteoarthritis and rheumatoid arthritis patients [52]. In response to endogenous or exogenous inflammatory cytokines (IL-1*β* and TNF-*α*), IKK*β* is activated first, leading to phosphorylation of I*κ*B*α* protein which degraded through ubiquitination which releases NF-*κ*B dimer into the nucleus binding to specific DNA sequences and promote transcription of inflammatory factors, chemokines, and metalloproteinases promoting the progression of osteoarthritis [53, 54]. Phosphoinositide-3 kinase (PI3K) consists of two subunits, p85 and P110, which regulate and catalyze the phosphorylation of the downstream Serine/threonine kinase AKT through lipid secondary messengers and regulate immune and inflammatory responses [55, 56]. More studies have shown that activation of NF-*κ*B pathway is often accompanied by activation of AKT. Inhibition of phosphorylation of PI3K/AKT pathway can partially block NF-*κ*B pathway and reduce transcription of downstream cytokines and protein-degrading enzymes [57, 58]. Our study also found that IL-1*β* stimulated chondrocytes increased the phosphorylation of PI3K and AKT and activated the NF-*κ*B pathway, while TFDG significantly inhibited the PI3K/AKT/NF-*κ*B pathway, thereby reducing the transcription of downstream inflammatory factors and metalloproteinases. Mitogen-activated protein kinase (MAPK) has three forms: C-Jun N-terminal kinases, P38 MAPKs, and ERKs. Among them, JNK and P38 MAPK pathways are activated mainly by receiving stimulation of inflammatory factors and signals of extracellular pressure [59, 60]. Many growth factors, cytokines, and bacterial metabolites can activate the ERKs pathway. Significantly increased IL-1*β* in synovium of patients with osteoarthritis can strongly activate C-Jun, P38 MAPKs, and ERKs through IL-1R. Previous studies have found that activation of MAPK pathway can increase the transcription of MMPs and ADAMTs and accelerate the degradation of extracellular matrix, while inhibitors of MAPK pathway can effectively protect the extracellular matrix and reduce chondrocyte apoptosis in vivo and in vitro [61, 62]. Similarly, we found that TFDG can effectively inhibit IL-1*β*-induced MAPK pathway activation by reducing c-Jun, P38 MAPKs, and ERKs phosphorylated proteins in chondrocytes.

**Figure 8:**
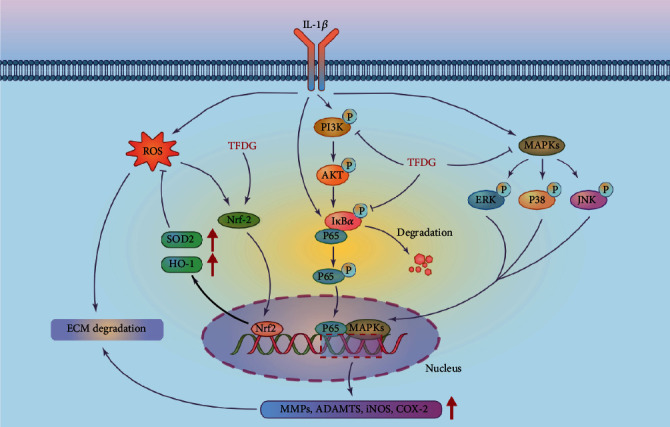
The mechanism of TFDG delaying cartilage degradation involves the activation of Nrf2/HO-1 signal pathway and the inhibition of PI3K/AKT/NF-*κ*B and MAPK pathways.

Our study found that TFDG could protect cartilage from degradation via its anti-inflammatory and antioxidant properties involving the enhancement of Nrf2/HO-1 signal pathway and the inhibition of PI3K/AKT/NF-*κ*B and MAPK pathways (Figure 8). However, our research has some limitations. Firstly, anti-inflammatory and antioxidant signal pathway may have crosstalk and we cannot separate them to analyze their function [63]. On the one hand, declined ROS which mediated by antioxidant enzymes can also reduce iNOS or COX2 protein expression and lessen the inflammatory reactions by decreased stimulation of NF-*κ*B pathway. HO-1, a downstream target gene of Nrf2, can even directly inhibited p65 nuclear translocation and NF-*κ*B mediated transcription including MMPs [64, 65]. On the other hand, the reduction of endogenous inflammatory factors due to the suppression of PI3K/AKT/NF-*κ*B and MAPK pathways decreased the production of ROS according to NOX4 complex, which also contributes to maintain redox homeostasis [66]. Secondly, we were unable to find specific agonist activating PI3K/AKT/NF-*κ*B and MAPK pathways to reverse the therapeutic effects of TFDG; thus, we cannot rule out interference between the pathways. Thirdly, we did not verify the inhibition of PI3K/AKT/NF-*κ*B and MAPK pathways in vivo via immunohistochemistry detection.

## 5. Conclusions

In conclusion, TFDG was found for the first time to exert anti-inflammatory effects in chondrocytes in vivo and vitro through the PI3K/AKT/NF-*κ*B and MAPK pathways and promote the antioxidant ability of chondrocytes through the Nrf2/HO-1 pathway. These results suggest a novel therapeutic approach for the treatment of OA.

## Data Availability

All data supporting the finding are available within the study.

## Abbreviations

OA: Osteoarthritis
IL-1: Interleukin 1
TNF: Tumor necrosis factor
ERK: Extracellular signal-regulated kinase
MAPK: Mitogen-activated protein kinase
JNK: C-Jun N-terminal kinase
NF-*κ*B: Nuclear factor kappa-B
I*κ*B*α*: Inhibitor kappa B alpha
PI3K: Phosphoinositide-3 kinase
AKT: Protein kinase B
ROS: Reactive oxygen species
TFDG: Theaflavin-3-3′-digallate
SOX9: SRY-related HMG box 9
COL-II: Type II collagen
ACAN: Aggrecan
MMP13: Matrixmetalloproteinase13
MMP3: Matrixmetalloproteinase3
ADAMTS5: A disintegrin and metalloproteinase with thrombospondin motifs5
Nrf2: Nuclear factor erythroid 2-related factor 2
HO-1: Heme oxygenase-1
SOD2: Superoxide dismutase2
CCK-8: Cell Counting Kit-8
PGE2: Prostaglandin E2
iNOS: Inducible nitric oxide synthase
COX2: Cyclooxygenase-2
ARE: Antioxidant response element.

## Supplementary Materials

Supplementary Materials Supplementary Table 1: the sequences used to detect target genes in the study were shown in the table.
