# Supplementary material for: Theaflavin-3,3′-Digallate Protects Cartilage from Degradation by Modulating Inflammation and Antioxidant Pathways
Source: Oxid Med Cell Longev. 2022 Jul 8;2022:3047425. doi: 10.1155/2022/3047425 (PMC9286955; doi:10.1155/2022/3047425)
Supplement: Supplementary Materials — Supplementary Table 1: the sequences used to detect target genes in the study were shown in the table. [file 3047425.f1.docx]

| gene | Forward | Backward |
| --- | --- | --- |
| *Rat-ACAN* | 5＇－CAACCTCCTGGGTGTAAGGA-3＇ | 5＇-GTGTAGCAGATGGCGTCGTA-3＇ |
| *Rat-Col2a1* | 5＇-TCCAGGTCTACAGGGAATGC-3＇ | 5＇-CTTTTCCAGGAGCTCCCTCT-3＇ |
| *Rat-MMP13* | 5＇-AAAGACTATCCCCGCCTCAT-3＇ | 5＇-TGGGCCCATTGAAAAAGTAG-3＇ |
| *Rat-SOX9* | 5＇-CATCAAGACGGAGCAACTGA-3＇ | 5＇-GTAGTGCGGAAGGTTGAGG-3＇ |
| *Rat-MMP3* | 5＇-CCACAGAATCCCCTGATGTC-3＇ | 5＇-CTGACTGCATCGAAGGACAA-3＇ |
| *Rat-TNF-α* | 5＇-GAGGTCAACCTGCCCAAGTA-3＇ | 5＇-GCTGGGTAGAGAACGGATGA-3＇ |
| *Rat-IL-6* | 5＇-CAGGAAGGCAGTGTCACTCA-3＇ | 5＇-AAAGAAGGTGCTTGGGTCCT-3＇ |
| *Rat-iNOS* | 5＇-AGACACATACTTTACGCCACTA-3＇ | 5＇-TCAAAGACCTCTGGATCTTGAC-3＇ |
| *Rat-Ptgs2* | 5＇-GAGCTGTAAGAGTCTACTGACC-3＇ | 5＇-ACACAGGAATCTTCACAAATGG-3＇ |
| *Rat-GAPDH* | 5＇-CCCCCAATGTATCCGTTGTG-3＇ | 5＇-TAGCCCAGGATGCCCTTTAGT-3＇ |
